# Telomere Signaling and Maintenance Pathways in Spermatozoa of Infertile Men Treated With Antioxidants: An *in silico* Approach Using Bioinformatic Analysis

**DOI:** 10.3389/fcell.2021.768510

**Published:** 2021-10-11

**Authors:** Manesh Kumar Panner Selvam, Saradha Baskaran, Suresh C. Sikka

**Affiliations:** Department of Urology, Tulane University Health Sciences Center, New Orleans, LA, United States

**Keywords:** antioxidants, bioinformatics, data mining, male infertility, sperm telomere, upstream regulators

## Abstract

Telomere shortening is considered as a marker of cellular senescence and it is regulated by various signaling pathways. Sperm telomere appears to play important role in its longevity and function. Antioxidant intake has been known to prevent the shortening of telomere. In the management of male infertility, antioxidants are commonly used to counterbalance the seminal oxidative stress. It is important to understand how antioxidants treatment may modulate telomere signaling in sperm. In the current study, we have identified 377 sperm proteins regulated by antioxidants based on data mining of published literature. Bioinformatic analysis revealed involvement of 399 upstream regulators and 806 master regulators associated with differentially expressed sperm proteins. Furthermore, upstream regulator analysis indicated activation of kinases (EGFR and MAPK3) and transcription factors (CCNE1, H2AX, MYC, RB1, and TP53). Hence, it is evident that antioxidant supplementation activates molecules associated with telomere function in sperm. The outcome of this *in silico* study suggests that antioxidant therapy has beneficial effects on certain transcription factors and kinases associated with sperm telomere maintenance and associated signaling pathways that may play an important role in the management of male factor infertility.

## Introduction

Telomere length (structures with non-coding hexanucleotide “TTAGGG” repeats) at the end of each chromosome determines its stability and genomic integrity. In human somatic (diploid) cells, telomere length is about 5 to 15 kb (Cross et al., 1989), whereas in germ cells (haploid) it is 10–15 kb (Samassekou et al., 2010; Ozturk, 2015). Telomere protects the chromosomal DNA from damage and is considered as a marker of cellular senescence (Bernadotte et al., 2016). Thus, telomere length maintenance is essential for normal cellular processes. Any abnormality in telomere length has been linked to age-related diseases as well as cancer (Stanley and Armanios, 2015).

In general, decrease in telomere length or telomere shortening adversely affects the functional characteristics of chromosomal DNA. Limited number of studies have focused on the role of sperm telomeres in reproduction and male infertility (Santana et al., 2019; Tahamtan et al., 2019; Darmishonnejad et al., 2020), of which few suggest that sperm telomere length (STL) is associated with sperm quality and DNA integrity (Ferlin et al., 2013; Cariati et al., 2016; Rocca et al., 2016; Darmishonnejad et al., 2020). Telomeres are highly rich in guanine and susceptible to oxidative damage (Coluzzi et al., 2014). *In vitro* studies suggest that oxidative stress accelerates telomere shortening and disrupts telomerase activity (Epel et al., 2004; Richter and von Zglinicki, 2007).

Increased oxidative stress associated with leukocytospermia is one of the prominent causes of male infertility and has deleterious effect on sperm DNA (Agarwal et al., 2019b). Moreover, sperm with poor chromatin protamination status are vulnerable to such oxidative attack (De Iuliis et al., 2009). Rocca et al. (2016) reported that protamination status of sperm chromatin is linked with STL (Rocca et al., 2016). Hence, defective chromatin packaging can increase the exposure of DNA to reactive oxygen species (ROS) resulting in telomere dysregulation in mature sperm.

Antioxidants that counterbalance the increased levels of seminal ROS are widely used in the management of oxidative stress-mediated male infertility (Agarwal et al., 2021a,b). Use of antioxidants in treatment of male infertility have shown to improve semen parameters (Keskes-Ammar et al., 2003; Smits et al., 2019; Arafa et al., 2020). Furthermore, antioxidant supplementation activates the molecular mechanism(s) associated with free radical scavenging in idiopathic infertile men and has positive beneficial effect on fertility associated sperm proteins (Agarwal et al., 2019a). In a cross-sectional study of children and adolescents, dietary antioxidants have been reported to reduce shortening of leukocyte telomere length (García-Calzón et al., 2015). However, the role of antioxidants in modulating sperm telomere signaling and maintenance is unknown. Therefore, the aim of this study is to review and conduct *in silico* analysis of omics data of sperm in patients subjected to antioxidant treatment to understand the effect of antioxidants on pathways involved in regulating STL in infertile men.

## Methods

**FIGURE 1:**
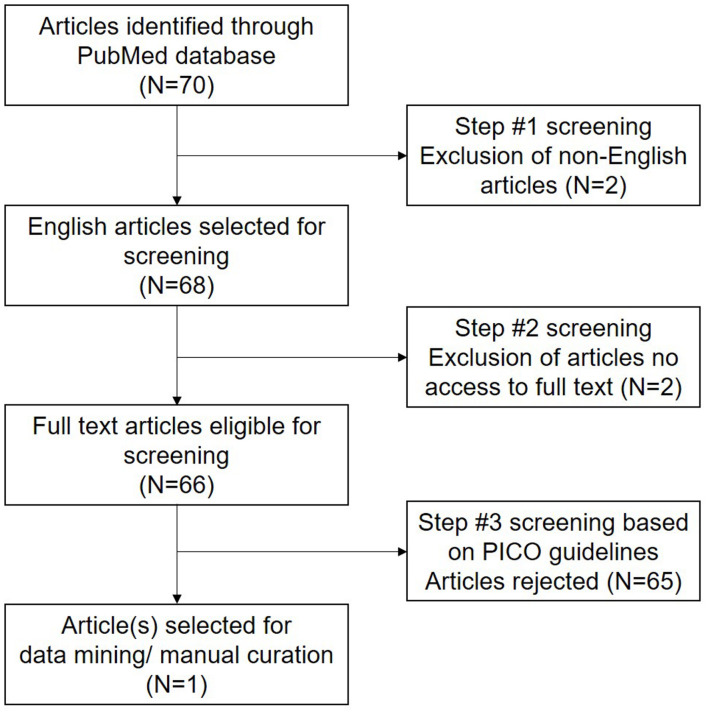
Preferred Reporting Items for Systematic Reviews and Meta-Analyses (PRISMA) workflow reporting the literature search strategy.

A comprehensive literature search was performed according to Preferred Reporting Items for Systematic Reviews and Meta-Analyses (PRISMA) guidelines. The articles were retrieved (Figure 1) from PubMed database on July 4, 2021 using the following string of keywords “(antioxidant^∗^ and sperm^∗^ and male infertility) and (proteomic^∗^ OR genomic^∗^ OR transcriptomic^∗^)”. Preliminary screening was carried out based on the following inclusion criteria: (a) studies conducted in humans, (b) involved antioxidant supplementation/treatment, and (c) reported laboratory evaluation of male infertility. Reviews, meta-analysis and studies not reporting clinical data were excluded. After preliminary screening, all the original studies were evaluated based on PICO (Population, Intervention, Control, and Outcome) guidelines (Supplementary Table 1).

Extensive data mining was carried out based on computational and manual approaches. The article (*n* = 1) in compliance with PICO guidelines was thoroughly searched for differentially expressed biomolecules reported in spermatozoa of infertile men. These annotated and curated biomolecules list containing gene/protein symbols with their respective expression values were saved as Microsoft Excel file. For further downstream analysis, this list was uploaded to ingenuity pathway analysis (IPA) software. Initially core analysis was conducted, and then casual network analysis was carried out to identify antioxidant activated kinases and transcription factors in sperm (Krämer et al., 2014). In-depth analysis was performed to identify those activated kinases and transcription factors that were either directly involved or linked with the molecules regulating telomere signaling pathway. Molecular Interaction Search Tool (MIST) was used to display interaction between the transcription factors and kinases associated with telomere signaling and maintenance pathway (Hu et al., 2018).

## Results and Discussion

Antioxidants are widely used in the treatment of male infertility. A recent global survey reported that 85.6% of physicians involved in the management of male infertility prescribe antioxidants as a part of their treatment regime (Agarwal et al., 2021a). Apart from improving the semen parameters, antioxidant intake increases the sperm DNA integrity without any side effects/complications (Zini et al., 2009; Majzoub et al., 2017; Arafa et al., 2020). Besides these benefits, antioxidants can delay the reduction of telomere length of somatic cells (Prasad et al., 2017). At subcellular level, antioxidants modulate proteins associated with CREM (cAMP responsive element modulator) signaling, mitochondrial function and protein oxidation (Agarwal et al., 2019a). They are also reported to activate antioxidant defense mechanism in sperm (Agarwal et al., 2019a). It is essential to understand the effect of antioxidant supplementation on mechanisms/pathways associated with sperm telomere. In the current study, we have used data mining and manual curation techniques to identify the molecules (sperm proteins) altered post-antioxidant treatment. For the first time, using an *in silico* approach this study sheds light on the beneficial role of antioxidants in regulating telomere signaling and maintenance pathways of sperm.

**FIGURE 2:**
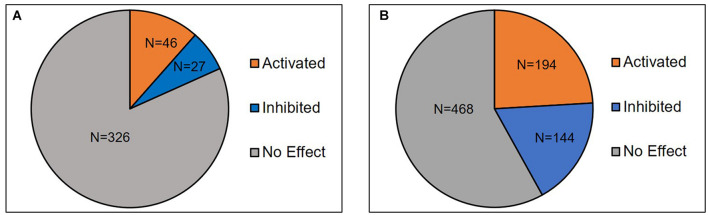
Distribution of **(A)** upstream regulators (*n* = 399) and **(B)** master regulators (*n* = 806) identified in the sperm post-antioxidant therapy.

Availability of different data mining strategies and accessibility to omics data made the researchers to reinvestigate the curated data with bioinformatic tools (Zhang and Chen, 2011; Alanis-Lobato, 2015). Such analysis led to the discovery of several existing and missing pathways linked to human diseases (Fechete et al., 2011; Narasimhan et al., 2014; Kharrat et al., 2019). Kothandaraman et al. (2016) used the data mining technique to identify genes associated with pathogenesis of idiopathic male infertility (Kothandaraman et al., 2016). In the current study, data mining and manual curation resulted in identification of 377 differentially expressed proteins in sperm following antioxidant therapy (Supplementary Table 2). Upstream regulator analysis (URA) revealed a total of 399 and 806 upstream regulators and master regulators, respectively. Upstream regulator analysis is an unique feature available in IPA to identify upstream regulators associated with differentially expressed genes/proteins (Li et al., 2015). Sperm proteomic studies have employed URA to identify regulatory molecules associated with reproductive function (Agarwal et al., 2019a; Panner Selvam et al., 2019). Figure 2 shows the distribution of 73 upstream regulators and 338 master regulators either activated (Z-score ≥ 2) or inhibited (Z-score ≤ –2) in our dataset. It is important to emphasize that none of the inhibited regulators were found to be involved in telomere function. Therefore, it clearly indicates that antioxidant supplementation has no negative effect on STL.

In-depth analysis revealed activation of kinases (EGFR: epidermal growth factor receptor and MAPK3: mitogen-activated protein kinase 3) associated with telomere function (Table 1). Epidermal growth factor receptor signaling pathway plays a pivotal role in regulation of telomere length via inhibiting telomerase activity (Maida et al., 2002; Tian et al., 2002; Augustine et al., 2017), whereas MAPK3/ERK2 pathway regulates telomeric repeat-binding factor 2 (TRF-2) to maintain telomere stability in a cell (Picco et al., 2016). In addition to kinases, using computational analysis we have also identified transcription factors (CCNE1: cyclin E1, H2AX: H2A.X variant histone, MYC: MYC proto-oncogene, RB1: RB transcriptional corepressor 1 and TP53: tumor protein p53) linked to the maintenance of telomere in sperm (Table 1). CCNE1 is mainly responsible for telomere stability (Martinerie et al., 2014), while absence of H2AX is linked to genomic instability (Celeste et al., 2002; Fernandez-Capetillo et al., 2003). Similarly, MYC regulates telomerase (Wang et al., 1998), particularly c-MYC interacts with TRF1/PIN2 (proteinase Inhibitor 2) leading to extension of telomere repeats (Kim and Chen, 2007).

**TABLE 1:** Transcription regulators activated in sperm after antioxidant therapy.

SN	Molecule	Category	Activation z-score	Telomere associated function(s)
1.	MYC	Transcription regulator	4.67	Telomere signaling, maintenance of telomere length
2.	CCNE1	Transcription regulator	3.04	Clustering of telomere
3.	MAPK3	Kinase	2.75	Modification of telomere length
4.	TP53	Transcription regulator	2.53	Telomere signaling, maintenance of telomere length
5.	RB1	Transcription regulator	2.35	Telomere signaling, maintenance of telomere length
6.	H2AX	Transcription regulator	2.09	Modification of telomere length
7.	EGFR	Kinase	2.06	Telomere signaling

**FIGURE 3:**
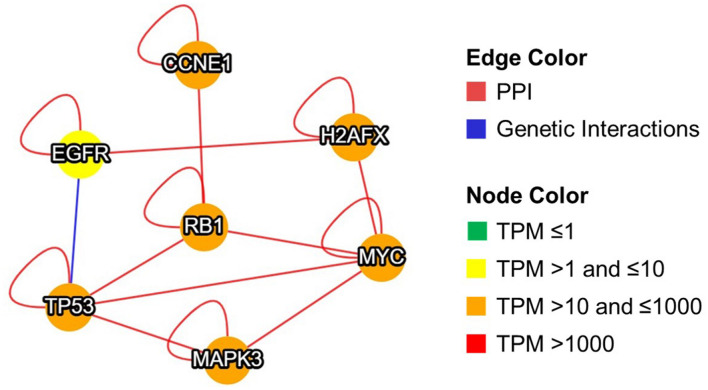
Interaction between transcription regulators and kinases involved in telomere signaling and maintenance pathway in sperm after antioxidant treatment. Graphical representation of network developed using MIST. PPI: protein-protein interaction, TPM: transcripts per million, MYC: MYC proto-oncogene, CCNE1: cyclin E1, MAPK3: mitogen-activated protein kinase 3, TP53: tumor protein p53, RB1: RB transcriptional corepressor 1, H2AX/H2AFX: H2A.X variant histone, EGFR: epidermal growth factor receptor.

Expression of RB1 proteins controls telomere length (García-Cao et al., 2002), while TP53 directly binds with chromosomal DNA and increases the stability of telomere (Tutton and Lieberman, 2017). Altered expression of these kinases and transcription factors may contribute toward telomere dysfunction in sperm of infertile men. Furthermore, MIST analysis displayed the interaction type (protein-protein or genetic) between the molecules (EGFR, MAPK3, CCNE1, H2AX, MYC, RB1, and TP53) and their abundance in the testis (Figure 3). New findings of this study clearly show that antioxidant supplementation activates the transcription regulators and kinases involved in sperm telomere signaling and maintenance pathway that may improve their longevity and function. Future clinical trials evaluating the STL post-antioxidant supplementation are warranted in infertile men to confirm its role in maintaining telomere integrity and sperm function. Such studies may provide more insight on the use of STL as a new prognostic or therapeutic marker of antioxidant effectiveness in the management of male infertility.

## Conclusion

For the first time, using bioinformatic approach, our results demonstrate that antioxidant therapy has positive effect on transcription factors and kinases associated with telomere function in sperm. Altered expression of EGFR, MAPK3, CCNE1, H2AX, MYC, RB1, and TP53 can serve as biomarkers for telomere dysfunction in sperm of infertile men, and opens new approaches to target improved therapies.

## Data Availability Statement

The original contributions presented in the study are included in the article/Supplementary Material, further inquiries can be directed to the corresponding authors.

## Author Contributions

MP conceived the idea and study design and conducted bioinformatic analysis. MP, SB, and SS wrote this article, reviewed, and approved the submitted version. All authors contributed to the article and approved the submitted version.

## Conflict of Interest

The authors declare that the research was conducted in the absence of any commercial or financial relationships that could be construed as a potential conflict of interest.

## Publisher’s Note

All claims expressed in this article are solely those of the authors and do not necessarily represent those of their affiliated organizations, or those of the publisher, the editors and the reviewers. Any product that may be evaluated in this article, or claim that may be made by its manufacturer, is not guaranteed or endorsed by the publisher.
