# Supplementary material for: Telomere Signaling and Maintenance Pathways in Spermatozoa of Infertile Men Treated With Antioxidants: An in silico Approach Using Bioinformatic Analysis
Source: Front Cell Dev Biol. 2021 Oct 11;9:768510. doi: 10.3389/fcell.2021.768510 (PMC8542908; doi:10.3389/fcell.2021.768510)
Supplement: Supplementary file 1 [file Table_1.pdf]

Table 1: PICO guidelines to select articles for data mining

Population	Human male patients
Intervention	Antioxidants
Control/Comparison	Untreated/ negative control or pre-treatment group
Outcome (Primary)	Expression of genes or transcripts or proteins
