# Supplementary material for: Telomere Signaling and Maintenance Pathways in Spermatozoa of Infertile Men Treated With Antioxidants: An in silico Approach Using Bioinformatic Analysis
Source: Front Cell Dev Biol. 2021 Oct 11;9:768510. doi: 10.3389/fcell.2021.768510 (PMC8542908; doi:10.3389/fcell.2021.768510)
Supplement: Supplementary file 2 [file Table_2.pdf]

Supplementary Table 2: Differentially expressed proteins in sperm after antioxidant treatment

SN	Symbol	Protein Name	Expression Fold Change	Location
1	A1BG	alpha-1-B glycoprotein	3.728	Extracellular Space
2	AASS	aminoadipate-semialdehyde synthase	5.106	Cytoplasm
3	ABCG2	ATP binding cassette subfamily G member 2 (Junior blood group)	∞	Plasma Membrane
4	ACADS	acyl-CoA dehydrogenase short chain	1.915	Cytoplasm
5	ACAT1	acetyl-CoA acetyltransferase 1	2.821	Cytoplasm
6	ACO2	aconitase 2	1.505	Cytoplasm
7	ACR	acrosin	-2.133	Extracellular Space
8	ACTG1	actin gamma 1	2.878	Cytoplasm
9	ACTR1A	actin related protein 1A	3.662	Cytoplasm
10	ACTRT3	actin related protein T3	-2.976	Nucleus
11	AGR2	anterior gradient 2, protein disulphide isomerase family member	2.936	Extracellular Space
12	AK1	adenylate kinase 1	4.189	Cytoplasm
13	ALDH1A1	aldehyde dehydrogenase 1 family member A1	10.322	Cytoplasm
14	ALDH1A2	aldehyde dehydrogenase 1 family member A2	∞	Cytoplasm
15	ANKRD28	ankyrin repeat domain 28	∞	Cytoplasm
16	ANXA3	annexin A3	-3.187	Cytoplasm
17	AP1B1	adaptor related protein complex 1 subunit beta 1	3.055	Cytoplasm
18	APMAP	adipocyte plasma membrane associated protein	-1.688	Plasma Membrane
19	APOA1	apolipoprotein A1	4.877	Extracellular Space
20	ARG1	arginase 1	-4.465	Cytoplasm
21	ARMC3	armadillo repeat containing 3	5.324	Cytoplasm

22	ARPC2	actin related protein 2/3 complex subunit 2	20.535	Cytoplasm
23	ARPC4	actin related protein 2/3 complex subunit 4	∞	Cytoplasm
24	ATP1A1	ATPase Na+/K+ transporting subunit alpha 1	2.153	Plasma Membrane
25	ATP1B1	ATPase Na+/K+ transporting subunit beta 1	-3.798	Plasma Membrane
26	ATP2B4	ATPase plasma membrane Ca2+ transporting 4	1.73	Plasma Membrane
27	ATP5F1A	ATP synthase F1 subunit alpha	1.809	Cytoplasm
28	ATP5F1B	ATP synthase F1 subunit beta	2.76	Cytoplasm
29	ATP5F1C	ATP synthase F1 subunit gamma	2.116	Cytoplasm
30	ATP5F1D	ATP synthase F1 subunit delta	8.047	Cytoplasm
31	ATP5MG	ATP synthase membrane subunit g	2.571	Cytoplasm
32	ATP5PD	ATP synthase peripheral stalk subunit d	2.82	Cytoplasm
33	ATP5PO	ATP synthase peripheral stalk subunit OSCP	2.194	Cytoplasm
34	ATP6V1A	ATPase H+ transporting V1 subunit A	3.29	Plasma Membrane
35	ATP6V1B2	ATPase H+ transporting V1 subunit B2	2.899	Cytoplasm
36	ATP6V1E2	ATPase H+ transporting V1 subunit E2	∞	Cytoplasm
37	ATP6V1G1	ATPase H+ transporting V1 subunit G1	3.626	Cytoplasm
38	AZGP1	alpha-2-glycoprotein 1, zinc-binding	2.079	Extracellular Space
39	BCAP31	B cell receptor associated protein 31	2.794	Cytoplasm
40	BPHL	biphenyl hydrolase like	13.56	Cytoplasm
41	C19orf18	chromosome 19 open reading frame 18	6.777	Cytoplasm
42	C2orf88	chromosome 2 open reading frame 88	22.315	Other
43	CAB39	calcium binding protein 39	4.502	Cytoplasm
44	CABYR	calcium binding tyrosine phosphorylation regulated	-1.913	Cytoplasm

45	CALR	calreticulin	1.725	Cytoplasm
46	CAMP	cathelicidin antimicrobial peptide	2.095	Cytoplasm
47	CAND1	cullin associated and neddylation dissociated 1	2.471	Cytoplasm
48	CAPZB	capping actin protein of muscle Z-line subunit beta	-2.801	Cytoplasm
49	CARM1	coactivator associated arginine methyltransferase 1	2.548	Nucleus
50	CARS1	cysteinyl-tRNA synthetase 1	6.224	Cytoplasm
51	CATSPERG	cation channel sperm associated auxiliary subunit gamma	-6.687	Extracellular Space
52	CCDC126	coiled-coil domain containing 126	3.719	Other
53	CCDC183	coiled-coil domain containing 183	∞	Other
54	CCDC90B	coiled-coil domain containing 90B	3.876	Cytoplasm
55	CCT2	chaperonin containing TCP1 subunit 2	2.235	Cytoplasm
56	CCT3	chaperonin containing TCP1 subunit 3	1.927	Cytoplasm
57	CCT4	chaperonin containing TCP1 subunit 4	2.377	Cytoplasm
58	CCT5	chaperonin containing TCP1 subunit 5	2.485	Cytoplasm
59	CCT7	chaperonin containing TCP1 subunit 7	1.938	Cytoplasm
60	CCT8	chaperonin containing TCP1 subunit 8	2.277	Cytoplasm
61	CCT6A	chaperonin containing TCP1 subunit 6A	2.92	Cytoplasm
62	CCT6B	chaperonin containing TCP1 subunit 6B	2.637	Cytoplasm
63	CD46	CD46 molecule	-2.982	Plasma Membrane
64	CD55	CD55 molecule (Cromer blood group)	-2.011	Plasma Membrane
65	CD177	CD177 molecule	-2.982	Cytoplasm
66	CDSN	corneodesmosin	-5.097	Plasma Membrane
67	CFAP20	cilia and flagella associated protein 20	-3.378	Cytoplasm
68	CFAP43	cilia and flagella associated protein 43	-2.14	Cytoplasm
69	CFAP52	cilia and flagella associated protein 52	-2.279	Cytoplasm

70	CLMN	calmin	3.582	Cytoplasm
71	CLPSL1	colipase like 1	-∞	Other
72	CLU	clusterin	2.358	Cytoplasm
73	CNP	2',3'-cyclic nucleotide 3' phosphodiesterase	-2.626	Cytoplasm
74	CNPY2	canopy FGF signaling regulator 2	2.708	Plasma Membrane
75	CPE	carboxypeptidase E	2.685	Cytoplasm
76	CPVL	carboxypeptidase vitellogenic like	3.696	Cytoplasm
77	CSNK2A1	casein kinase 2 alpha 1	-2.414	Nucleus
78	CSNK2B	casein kinase 2 beta	-3.572	Cytoplasm
79	CYC1	cytochrome c1	-1.712	Cytoplasm
80	CYCS	cytochrome c, somatic	2.886	Cytoplasm
81	DARS2	aspartyl-tRNA synthetase 2, mitochondrial	13.44	Cytoplasm
82	DCTN1	dynactin subunit 1	3.075	Cytoplasm
83	DCTN2	dynactin subunit 2	6.767	Cytoplasm
84	DCTN3	dynactin subunit 3	3.2	Nucleus
85	DCUN1D1	defective in cullin neddylation 1 domain containing 1	4.16	Nucleus
86	DDT	D-dopachrome tautomerase	3.593	Cytoplasm
87	DECR1	2,4-dienoyl-CoA reductase 1	1.594	Cytoplasm
88	DHRS7B	dehydrogenase/reductase 7B	3.377	Other
89	DLAT	dihydrolipoamide S-acetyltransferase	-2.606	Cytoplasm
90	DLST	dihydrolipoamide S-succinyltransferase	-3.053	Cytoplasm
91	DNAJB6	DnaJ heat shock protein family (Hsp40) member B6	-3.963	Nucleus
92	DNAJC3	DnaJ heat shock protein family (Hsp40) member C3	4.5	Cytoplasm
93	DNALI1	dynein axonemal light intermediate chain 1	2.068	Cytoplasm

94	DPCD	deleted in primary ciliary dyskinesia homolog (mouse)	2.717	Nucleus
95	DSC3	desmocollin 3	-3.988	Plasma Membrane
96	DSG1	desmoglein 1	-2.757	Plasma Membrane
97	DSP	desmoplakin	-2.296	Plasma Membrane
98	DUT	deoxyuridine triphosphatase	1.998	Nucleus
99	DYNLRB2	dynein light chain roadblock-type 2	2.529	Cytoplasm
100	ECI1	enoyl-CoA delta isomerase 1	1.997	Cytoplasm
101	EEF2	eukaryotic translation elongation factor 2	-2.096	Cytoplasm
102	EEF1E1	eukaryotic translation elongation factor 1 epsilon 1	7.388	Cytoplasm
103	EEF1G	eukaryotic translation elongation factor 1 gamma	-1.688	Cytoplasm
104	EFCAB1	EF-hand calcium binding domain 1	44.361	Cytoplasm
105	EFCAB6	EF-hand calcium binding domain 6	-3.121	Nucleus
106	EFHC1	EF-hand domain containing 1	-11.464	Cytoplasm
107	EFHC2	EF-hand domain containing 2	-12.608	Cytoplasm
108	EIF3A	eukaryotic translation initiation factor 3 subunit A	∞	Cytoplasm
109	EIF3F	eukaryotic translation initiation factor 3 subunit F	2.212	Cytoplasm
110	EIF3M	eukaryotic translation initiation factor 3 subunit M	4.086	Cytoplasm
111	ELSPBP1	epididymal sperm binding protein 1	-1.996	Other
112	ERP29	endoplasmic reticulum protein 29	3.11	Cytoplasm
113	ETFA	electron transfer flavoprotein subunit alpha	1.792	Cytoplasm
114	ETFB	electron transfer flavoprotein subunit beta	4.944	Cytoplasm
115	ETFDH	electron transfer flavoprotein dehydrogenase	5.059	Cytoplasm

116	ETFRF1	electron transfer flavoprotein regulatory factor 1	5.106	Cytoplasm
117	EZR	ezrin	2.917	Plasma Membrane
118	FAAH	fatty acid amide hydrolase	-1.847	Plasma Membrane
119	FAM71B	family with sequence similarity 71 member B	-3.614	Nucleus
120	FARSB	phenylalanyl-tRNA synthetase subunit beta	5.962	Cytoplasm
121	FDXR	ferredoxin reductase	9.564	Cytoplasm
122	FGL1	fibrinogen like 1	-2.664	Extracellular Space
123	FH	fumarate hydratase	2.101	Cytoplasm
124	FKBP2	FKBP prolyl isomerase 2	2.829	Cytoplasm
125	FLAD1	flavin adenine dinucleotide synthetase 1	13.128	Cytoplasm
126	FLNB	filamin B	-2.114	Cytoplasm
127	FMR1NB	FMR1 neighbor	-2.04	Nucleus
128	FUCA1	alpha-L-fucosidase 1	2.332	Cytoplasm
129	GANAB	glucosidase II alpha subunit	4.461	Cytoplasm
130	GAPDHS	glyceraldehyde-3-phosphate dehydrogenase, spermatogenic	-13.426	Cytoplasm
131	GATD3A/GATD3B	glutamine amidotransferase like class 1 domain containing 3A	4.935	Cytoplasm
132	GDE1	glycerophosphodiester phosphodiesterase 1	-6.355	Plasma Membrane
133	GDPD1	glycerophosphodiester phosphodiesterase domain containing 1	-2.457	Cytoplasm
134	GFPT1	glutamine--fructose-6-phosphate transaminase 1	-2.779	Cytoplasm
135	GK2	glycerol kinase 2	-2.619	Cytoplasm
136	GK3P	glycerol kinase 3 pseudogene	-2.95	Other
137	GLB1L	galactosidase beta 1 like	-2.14	Extracellular Space
138	GLIPR1L2	GLIPR1 like 2	-2.697	Extracellular Space

139	GLRX5	glutaredoxin 5	4.112	Cytoplasm
140	GLUL	glutamate-ammonia ligase	-10.422	Cytoplasm
141	GOLT1B	golgi transport 1B	2.182	Cytoplasm
142	GPD2	glycerol-3-phosphate dehydrogenase 2	-2.271	Cytoplasm
143	GPI	glucose-6-phosphate isomerase	1.552	Extracellular Space
144	GPX4	glutathione peroxidase 4	-2.646	Cytoplasm
145	GRPEL1	GrpE like 1, mitochondrial	4.691	Cytoplasm
146	GSDMA	gasdermin A	-11.342	Cytoplasm
147	GSTO2	glutathione S-transferase omega 2	-6.04	Cytoplasm
148	GSTZ1	glutathione S-transferase zeta 1	2.78	Cytoplasm
149	H2AB3 (includes others)	H2A.B variant histone 3	6.771	Nucleus
150	H2AZ2	H2A.Z variant histone 2	4.19	Nucleus
151	H2BC1	H2B clustered histone 1	2.119	Nucleus
152	HADH	hydroxyacyl-CoA dehydrogenase	1.495	Cytoplasm
153	HDDC2	HD domain containing 2	38.431	Cytoplasm
154	HDHD5	haloacid dehalogenase like hydrolase domain containing 5	4.322	Cytoplasm
155	HIGD1A	HIG1 hypoxia inducible domain family member 1A	2.42	Cytoplasm
156	HIGD2A	HIG1 hypoxia inducible domain family member 2A	3.537	Cytoplasm
157	HINT2	histidine triad nucleotide binding protein 2	2.067	Cytoplasm
158	HK1	hexokinase 1	2.682	Cytoplasm
159	HMOX2	heme oxygenase 2	2.282	Cytoplasm
160	HPRT1	hypoxanthine phosphoribosyltransferase 1	2.154	Cytoplasm
161	HSCB	HscB mitochondrial iron-sulfur cluster cochaperone	12.912	Cytoplasm

162	HSD17B10	hydroxysteroid 17-beta dehydrogenase 10	1.704	Cytoplasm
163	HSP90B1	heat shock protein 90 beta family member 1	2.277	Cytoplasm
164	HSPA4	heat shock protein family A (Hsp70) member 4	4.081	Cytoplasm
165	HSPA13	heat shock protein family A (Hsp70) member 13	10.644	Cytoplasm
166	HSPA4L	heat shock protein family A (Hsp70) member 4 like	3.8	Cytoplasm
167	HSPD1	heat shock protein family D (Hsp60) member 1	3.258	Cytoplasm
168	HSPE1	heat shock protein family E (Hsp10) member 1	2.031	Cytoplasm
169	HSPH1	heat shock protein family H (Hsp110) member 1	3.432	Cytoplasm
170	HYOU1	hypoxia up-regulated 1	2.924	Cytoplasm
171	IDH1	isocitrate dehydrogenase (NADP(+)) 1	4.772	Cytoplasm
172	IDH2	isocitrate dehydrogenase (NADP(+)) 2	2.779	Cytoplasm
173	IDH3B	isocitrate dehydrogenase (NAD(+)) 3 non-catalytic subunit beta	3.207	Cytoplasm
174	IL4I1	interleukin 4 induced 1	1.832	Cytoplasm
175	IMMP2L	inner mitochondrial membrane peptidase subunit 2	3.027	Cytoplasm
176	IQCN	IQ motif containing N	-2.213	Cytoplasm
177	IQGAP2	IQ motif containing GTPase activating protein 2	-∞	Cytoplasm
178	ISOC2	isochorismatase domain containing 2	1.583	Cytoplasm
179	JUP	junction plakoglobin	-2.206	Plasma Membrane
180	KLHL10	kelch like family member 10	-2.292	Nucleus
181	KPNB1	karyopherin subunit beta 1	1.577	Nucleus
182	KPRP	keratinocyte proline rich protein	-2.347	Cytoplasm

183	KRT1	keratin 1	-2.526	Cytoplasm
184	KRT2	keratin 2	-2.668	Cytoplasm
185	KRT9	keratin 9	-2.438	Cytoplasm
186	KRT18	keratin 18	∞	Cytoplasm
187	KTN1	kinectin 1	2.352	Plasma Membrane
188	L2HGDH	L-2-hydroxyglutarate dehydrogenase	∞	Cytoplasm
189	LDHB	lactate dehydrogenase B	6.549	Cytoplasm
190	LDHC	lactate dehydrogenase C	3.119	Cytoplasm
191	LGMN	legumain	6.428	Cytoplasm
192	LONP1	lon peptidase 1, mitochondrial	3.597	Cytoplasm
193	LYRM7	LYR motif containing 7	18.675	Cytoplasm
194	MANF	mesencephalic astrocyte derived neurotrophic factor	2.802	Extracellular Space
195	MCCC2	methylcrotonoyl-CoA carboxylase 2	8.353	Cytoplasm
196	ME1	malic enzyme 1	2.867	Cytoplasm
197	ME2	malic enzyme 2	-1.671	Cytoplasm
198	MIF	macrophage migration inhibitory factor	∞	Extracellular Space
199	MLF1	myeloid leukemia factor 1	-5.604	Nucleus
200	MMAA	metabolism of cobalamin associated A	∞	Cytoplasm
201	MMAB	metabolism of cobalamin associated B	6.188	Cytoplasm
202	MME	membrane metalloendopeptidase	-1.734	Plasma Membrane
203	MPST	mercaptopyruvate sulfurtransferase	8.299	Cytoplasm
204	MPV17	mitochondrial inner membrane protein MPV17	-5.872	Cytoplasm
205	MT-CO1	cytochrome c oxidase subunit I	-2.977	Cytoplasm
206	MTX1	metaxin 1	2.523	Cytoplasm
207	MXRA5	matrix remodeling associated 5	7.919	Extracellular Space
208	MYDGF	myeloid derived growth factor	3.314	Extracellular Space

209	NAPA	NSF attachment protein alpha	2.609	Cytoplasm
210	NAXD	NAD(P)HX dehydratase	6.981	Cytoplasm
211	NDUFA6	NADH:ubiquinone oxidoreductase subunit A6	2.703	Cytoplasm
212	NDUFA9	NADH:ubiquinone oxidoreductase subunit A9	2.487	Cytoplasm
213	NDUFA13	NADH:ubiquinone oxidoreductase subunit A13	3.301	Cytoplasm
214	NDUFB5	NADH:ubiquinone oxidoreductase subunit B5	2.988	Cytoplasm
215	NDUFB11	NADH:ubiquinone oxidoreductase subunit B11	-5.56	Cytoplasm
216	NDUFS1	NADH:ubiquinone oxidoreductase core subunit S1	1.828	Cytoplasm
217	NDUFS8	NADH:ubiquinone oxidoreductase core subunit S8	3.697	Cytoplasm
218	NIPSNAP3A	nipsnap homolog 3A	-1.629	Nucleus
219	NME7	NME/NM23 family member 7	-14.92	Cytoplasm
220	NONO	non-POU domain containing octamer binding	$-\infty$	Nucleus
221	NPEPPS	aminopeptidase puromycin sensitive	4.157	Cytoplasm
222	NPTX2	neuronal pentraxin 2	$-\infty$	Extracellular Space
223	NT5C	5', 3'-nucleotidase, cytosolic	5.802	Cytoplasm
224	NUP35	nucleoporin 35	5.442	Nucleus
225	NUP58	nucleoporin 58	2.11	Nucleus
226	NUP155	nucleoporin 155	-2.136	Nucleus
227	NUP205	nucleoporin 205	-2.075	Nucleus
228	NUP210	nucleoporin 210	-13.334	Nucleus
229	NUP210L	nucleoporin 210 like	-30.144	Other
230	OXCT1	3-oxoacid CoA-transferase 1	3.27	Cytoplasm
231	P4HB	prolyl 4-hydroxylase subunit beta	1.536	Cytoplasm

232	PARK7	Parkinsonism associated deglycase	2.694	Nucleus
233	PCK2	phosphoenolpyruvate carboxykinase 2, mitochondrial	∞	Cytoplasm
234	PDHB	pyruvate dehydrogenase E1 subunit beta	1.958	Cytoplasm
235	PDIA3	protein disulfide isomerase family A member 3	2.439	Cytoplasm
236	PDIA4	protein disulfide isomerase family A member 4	1.813	Cytoplasm
237	PDIA6	protein disulfide isomerase family A member 6	1.747	Cytoplasm
238	PDILT	protein disulfide isomerase like, testis expressed	3.687	Extracellular Space
239	PEX11B	peroxisomal biogenesis factor 11 beta	2.751	Cytoplasm
240	PFKP	phosphofructokinase, platelet	1.696	Cytoplasm
241	PGAM5	PGAM family member 5, mitochondrial serine/threonine protein phosphatase	2.55	Cytoplasm
242	PGP	phosphoglycolate phosphatase	4.263	Cytoplasm
243	PGS1	phosphatidylglycerophosphate synthase 1	∞	Cytoplasm
244	PHB2	prohibitin 2	3.32	Cytoplasm
245	PHB	prohibitin	3.816	Nucleus
246	PIP	prolactin induced protein	2.096	Extracellular Space
247	PITRM1	pitrilysin metallopeptidase 1	1.873	Cytoplasm
248	PKM	pyruvate kinase M1/2	-1.835	Cytoplasm
249	PKP1	plakophilin 1	-3.445	Plasma Membrane
250	PLIN3	perilipin 3	∞	Cytoplasm
251	PMPCA	peptidase, mitochondrial processing subunit alpha	6.031	Cytoplasm
252	PMPCB	peptidase, mitochondrial processing subunit beta	2.776	Cytoplasm
253	PPIL6	peptidylprolyl isomerase like 6	-3.352	Other
254	PPOX	protoporphyrinogen oxidase	7.584	Cytoplasm

255	PPP2R1A	protein phosphatase 2 scaffold subunit Aalpha	3.551	Cytoplasm
256	PPP4R1	protein phosphatase 4 regulatory subunit 1	3.836	Other
257	PRDX5	peroxiredoxin 5	2.775	Cytoplasm
258	PRKACA	protein kinase cAMP-activated catalytic subunit alpha	2.53	Cytoplasm
259	PRKAR1A	protein kinase cAMP-dependent type I regulatory subunit alpha	3.676	Cytoplasm
260	PRKAR2A	protein kinase cAMP-dependent type II regulatory subunit alpha	1.674	Cytoplasm
261	PRKCSH	protein kinase C substrate 80K-H	3.012	Cytoplasm
262	PRPS2	phosphoribosyl pyrophosphate synthetase 2	∞	Cytoplasm
263	PSMB1	proteasome 20S subunit beta 1	2.096	Cytoplasm
264	PSMB2	proteasome 20S subunit beta 2	2.501	Cytoplasm
265	PSMB3	proteasome 20S subunit beta 3	2.045	Cytoplasm
266	PSMD3	proteasome 26S subunit, non-ATPase 3	2.365	Cytoplasm
267	PSMD4	proteasome 26S subunit, non-ATPase 4	2.929	Cytoplasm
268	PSMD6	proteasome 26S subunit, non-ATPase 6	3.271	Cytoplasm
269	PSMD11	proteasome 26S subunit, non-ATPase 11	2.44	Cytoplasm
270	PSMD13	proteasome 26S subunit, non-ATPase 13	2.066	Cytoplasm
271	PSME2	proteasome activator subunit 2	11.193	Cytoplasm
272	PTPMT1	protein tyrosine phosphatase mitochondrial 1	3.484	Cytoplasm
273	PTRHD1	peptidyl-tRNA hydrolase domain containing 1	9.523	Cytoplasm
274	RAB14	RAB14, member RAS oncogene family	2.847	Cytoplasm
275	RAB11B	RAB11B, member RAS oncogene family	3.307	Cytoplasm
276	RAB5B	RAB5B, member RAS oncogene family	∞	Cytoplasm
277	RAB5C	RAB5C, member RAS oncogene family	3.852	Cytoplasm

278	RAB6A	RAB6A, member RAS oncogene family	2.894	Cytoplasm
279	RARS1	arginyl-tRNA synthetase 1	8.676	Cytoplasm
280	REEP6	receptor accessory protein 6	2.306	Plasma Membrane
281	RNF121	ring finger protein 121	-11.056	Other
282	RPL3	ribosomal protein L3	2.712	Nucleus
283	RPL5	ribosomal protein L5	2.587	Cytoplasm
284	RPL6	ribosomal protein L6	2.01	Nucleus
285	RPL8	ribosomal protein L8	∞	Cytoplasm
286	RPL9	ribosomal protein L9	3.55	Nucleus
287	RPL11	ribosomal protein L11	2.657	Cytoplasm
288	RPL12	ribosomal protein L12	2.541	Nucleus
289	RPL13	ribosomal protein L13	14.37	Nucleus
290	RPL14	ribosomal protein L14	4.929	Cytoplasm
291	RPL17	ribosomal protein L17	∞	Cytoplasm
292	RPL18	ribosomal protein L18	18.192	Cytoplasm
293	RPL28	ribosomal protein L28	57.861	Cytoplasm
294	RPL31	ribosomal protein L31	9.496	Cytoplasm
295	RPL36	ribosomal protein L36	14.931	Cytoplasm
296	RPL38	ribosomal protein L38	∞	Cytoplasm
297	RPL10A	ribosomal protein L10a	2.263	Nucleus
298	RPL13A	ribosomal protein L13a	4.052	Cytoplasm
299	RPS3	ribosomal protein S3	2.741	Cytoplasm
300	RPS5	ribosomal protein S5	21.492	Cytoplasm
301	RPS7	ribosomal protein S7	5.996	Cytoplasm
302	RPS10	ribosomal protein S10	32.134	Cytoplasm
303	RPS12	ribosomal protein S12	4.191	Cytoplasm
304	RPS13	ribosomal protein S13	3.558	Cytoplasm
305	RPS16	ribosomal protein S16	4.006	Cytoplasm

306	RPS18	ribosomal protein S18	3.729	Cytoplasm
307	RPS19	ribosomal protein S19	4.946	Cytoplasm
308	RPS20	ribosomal protein S20	23.972	Cytoplasm
309	RPS21	ribosomal protein S21	∞	Cytoplasm
310	RPSA	ribosomal protein SA	3.586	Cytoplasm
311	RSPH1	radial spoke head component 1	-3.344	Nucleus
312	RSPH9	radial spoke head component 9	-1.939	Cytoplasm
313	RSPH6A	radial spoke head 6 homolog A	-3.607	Extracellular Space
314	RUVBL2	RuvB like AAA ATPase 2	1.733	Nucleus
315	SACM1L	SAC1 like phosphatidylinositide phosphatase	2.527	Cytoplasm
316	SAMM50	SAMM50 sorting and assembly machinery component	-4.617	Cytoplasm
317	SARAF	store-operated calcium entry associated regulatory factor	-35.42	Cytoplasm
318	SCCPDH	saccharopine dehydrogenase (putative)	-1.63	Cytoplasm
319	SDR39U1	short chain dehydrogenase/reductase family 39U member 1	5.24	Nucleus
320	SEC61B	SEC61 translocon subunit beta	3.811	Cytoplasm
321	SELENOI	selenoprotein I	-3.264	Cytoplasm
322	SEMG1	semenogelin 1	2.074	Extracellular Space
323	SEMG2	semenogelin 2	2.977	Extracellular Space
324	SERPINB12	serpin family B member 12	-4.276	Cytoplasm
325	SH3GLB1	SH3 domain containing GRB2 like, endophilin B1	2.81	Cytoplasm
326	SLC25A10	solute carrier family 25 member 10	2.079	Cytoplasm
327	SLC25A42	solute carrier family 25 member 42	3.025	Cytoplasm
328	SLC26A3	solute carrier family 26 member 3	22.144	Plasma Membrane
329	SLC2A14	solute carrier family 2 member 14	2.021	Nucleus
330	SLC44A5	solute carrier family 44 member 5	-2.291	Plasma Membrane

331	SMPD4	sphingomyelin phosphodiesterase 4	-2.576	Cytoplasm
332	SPA17	sperm autoantigenic protein 17	8.814	Plasma Membrane
333	SPACA1	sperm acrosome associated 1	-2.325	Cytoplasm
334	SPACA9	sperm acrosome associated 9	2.646	Cytoplasm
335	SPACA5/ SPACA5B	sperm acrosome associated 5B	2.732	Other
336	SPCS1	signal peptidase complex subunit 1	-3.434	Cytoplasm
337	SPESP1	sperm equatorial segment protein 1	3.885	Cytoplasm
338	SSNA1	SS nuclear autoantigen 1	11.295	Cytoplasm
339	ST13	ST13 Hsp70 interacting protein	4.128	Cytoplasm
340	STIP1	stress induced phosphoprotein 1	2.613	Cytoplasm
341	STX12	syntaxin 12	2.845	Cytoplasm
342	STYXL1	serine/threonine/tyrosine interacting like 1	-4.12	Cytoplasm
343	SYT7	synaptotagmin 7	-2.75	Cytoplasm
344	TCP1	t-complex 1	2.305	Cytoplasm
345	TEX44	testis expressed 44	3.019	Cytoplasm
346	THNSL1	threonine synthase like 1	∞	Nucleus
347	THSD4	thrombospondin type 1 domain containing 4	-3.715	Cytoplasm
348	TIMM17B	translocase of inner mitochondrial membrane 17B	∞	Cytoplasm
349	TKFC	triokinase and FMN cyclase	3.315	Cytoplasm
350	TKTL1	transketolase like 1	3.39	Cytoplasm
351	TM9SF3	transmembrane 9 superfamily member 3	-1.992	Cytoplasm
352	TMCO2	transmembrane and coiled-coil domains 2	2.152	Nucleus
353	TMEM89	transmembrane protein 89	-3.068	Nucleus
354	TMEM160	transmembrane protein 160	-7.409	Cytoplasm
355	TMEM205	transmembrane protein 205	-2.144	Cytoplasm

356	TMEM126A	transmembrane protein 126A	2.115	Cytoplasm
357	TMEM38B	transmembrane protein 38B	-5.322	Nucleus
358	TOMM20	translocase of outer mitochondrial membrane 20	4.248	Cytoplasm
359	TOMM34	translocase of outer mitochondrial membrane 34	∞	Cytoplasm
360	TOR2A	torsin family 2 member A	4.94	Extracellular Space
361	TPI1	triosephosphate isomerase 1	-17.247	Cytoplasm
362	TPP2	tripeptidyl peptidase 2	5.303	Cytoplasm
363	TPPP2	tubulin polymerization promoting protein family member 2	∞	Nucleus
364	TSPAN16	tetraspanin 16	-2.087	Other
365	TUBB3	tubulin beta 3 class III	-2.771	Cytoplasm
366	TUBB8	tubulin beta 8 class VIII	-∞	Cytoplasm
367	TUFM	Tu translation elongation factor, mitochondrial	16.531	Cytoplasm
368	TXNDC17	thioredoxin domain containing 17	15.261	Cytoplasm
369	UCHL1	ubiquitin C-terminal hydrolase L1	12.932	Cytoplasm
370	UCHL3	ubiquitin C-terminal hydrolase L3	2.544	Cytoplasm
371	UQCRB	ubiquinol-cytochrome c reductase binding protein	2.04	Cytoplasm
372	VAMP3	vesicle associated membrane protein 3	2.155	Plasma Membrane
373	VCP	valosin containing protein	1.924	Cytoplasm
374	VPS13A	vacuolar protein sorting 13 homolog A	1.644	Cytoplasm
375	WBP2NL	WBP2 N-terminal like	-3.213	Cytoplasm
376	ZDHHC3	zinc finger DHHC-type palmitoyltransferase 3	-7.647	Cytoplasm
377	ZPBP	zona pellucida binding protein	-1.567	Nucleus

Note: *These data were extracted from Agarwal, Panner Selvam et al., 2019 and subjected to bioinformatic analysis using ingenuity pathway analysis tool. Study subjects: idiopathic infertile men; antioxidant formulation: FH PRO for Men antioxidant capsules (1000 µg B12, 30 mg zinc, 140 µg selenium, 350 mg l-arginine, 2000 mg l-carnitine tartrate, 200 mg Co-Q10, 120 mg vitamin C, 200 IU vitamin E); dose and treatment duration: 3 capsules/day for a period of 6 months.* ∞*: Expressed in sperm only after antioxidant treatment;* -∞*: Absent in sperm after antioxidant treatment.*
